# *N*-n-butyl haloperidol iodide protects cardiomyocytes against hypoxia/reoxygenation injury by inhibiting autophagy

**DOI:** 10.18632/oncotarget.5077

**Published:** 2015-09-02

**Authors:** Bin Wang, Shuping Zhong, Fuchun Zheng, Yanmei Zhang, Fenfei Gao, Yicun Chen, Binger Lu, Han Xu, Ganggang Shi

**Affiliations:** ^1^ Department of Pharmacology, Shantou University Medical College, Shantou, 515041 Guangdong, China; ^2^ Department of Biochemistry and Molecular Biology, University of Southern California, Los Angeles, CA 90033, USA; ^3^ Department of Pharmacy, The First Affiliated Hospital, Shantou University Medical College, Shantou, 515041 Guangdong, China; ^4^ Department of Cardiovascular Diseases, The First Affiliated Hospital, Shantou University Medical College, Shantou, 515041 Guangdong, China

**Keywords:** Pathology Section, autophagy, *N*-n-butyl haloperidol iodide, hypoxia/reoxygenation, apoptosis, cardiomyocytes

## Abstract

*N*-n-butyl haloperidol iodide (F_2_), a novel compound derived from haloperidol, protects against the damaging effects of ischemia/reperfusion (I/R) injury *in vitro* and *in vivo*. In this study, we hypothesized the myocardial protection of F_2_ on cardiomyocyte hypoxia/reoxygenation (H/R) injury is mediated by inhibiting autophagy in H9c2 cells. The degree of autophagy by treatment with F_2_ exposed to H/R in H9c2 cell was characterized by monodansylcadaverine, transmission electron microscopy, and expression of autophagy marker protein LC3. Our results indicated that treatment with F_2_ inhibited autophagy in H9c2 cells exposed to H/R. 3-methyladenine, an inhibitor of autophagy, suppressed H/R-induced autophagy, and decreased apoptosis, whereas rapamycin, a classical autophagy sensitizer, increased autophagy and apoptosis. Mechanistically, macrophage migration inhibitory factor (MIF) was inhibited by F_2_ treatment after H/R. Accordingly, small interfering RNA (siRNA)-mediated MIF knockdown decreased H/R-induced autophagy. In summary, F2 protects cardiomyocytes during H/R injury through suppressing autophagy activation. Our results provide a new mechanistic insight into a functional role of F_2_ against H/R-induced cardiomyocyte injury and death.

## INTRODUCTION

Exacerbated tissue and organ damage produced by the restoration of blood flow after ischemia is known as ischemia/reperfusion (I/R) injury. Myocardial I/R injury is a pathophysiological phenomenon commonly seen after ischemia heart disease and heart surgery. I/R injury is caused by disturbances in ionic homeostasis, local edema, free radical stress and inflammatory responses. Recently, many studies report that autophagy also plays a key role in myocardial I/R injury in both animal models and cellular models by causing progressive degeneration of the heart [1]. Autophagy is a dynamic process that turns over organelles and proteins through a lysosome-associated degradation process, and serves a critical function in cellular homoeostasis by regulating cell survival and cell death pathways [2]. Autophagy has been implicated in myocardial I/R. However, the role of autophagy in contributing to cell survival and death in the heart remains controversial [3, 4]. Several lines of evidence suggest that autophagy may promote cell survival by purging the cell of damaged proteins to generate the intracellular building blocks required to maintain vital functions during nutrient-limiting conditions, such as ischemia or hypoxia [5, 6]. However, most lines of evidence suggest that up-regulation of autophagy during hypoxia/reoxygenation (H/R) or I/R may be detrimental [7, 8]. Autophagy may promote cell death through excessive self-digestion and degradation of essential cellular constituents. Many damaged cardiomyocytes show features of autophagic/lysosomal cell death during myocardial I/R injury, including the appearance of cytoplasmic autophagic vacuoles and the recruitment of LC3 to autophagosomes [9]. Inhibition of H/R-induced autophagy by 3-methyladenine (3-MA) or knockdown of Beclin 1 leads to enhanced cardiac myocyte survival *in vitro* [10]. In addition, inhibition of autophagy reduces the size of myocardial infarction and cardiac myocyte apoptosis in *beclin 1^+/−^* mice [4].

Moderate autophagy may promote cell survival while the excessive autophagy can accelerate cell death. As such, autophagy as a potential target for the treatment of ischemic heart diseases has become of increasing interest. Furthermore, it will provide a new therapeutic strategy for the prevention of I/R injury. New drugs that regulate cardiomyocyte autophagy will enhance our ability to ameliorate myocardial I/R injury and aid in the investigation of the role of autophagy in the pathogenesis of I/R injury.

**Figure 1:**
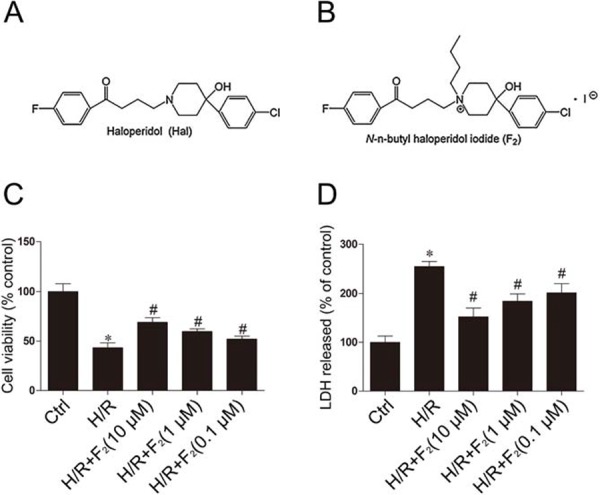
F_2_ promotes cell survival and reduces cell damage after H/R in myocardial H9c2 cells **A.** Chemical structure of haloperidol (Hal). **B.** Chemical structure of *N*-n-butyl haloperidol iodide (F_2_). **C.** Cell viability was measured by MTT assay. **D.** The release of LDH in culture medium was determined at the end of reoxygenation. The data shown are represented as the means ± SD confirmed in three separate experiments. *p < 0.05 vs. control, #*p* < 0.05 vs. H/R. Ctrl: control; H/R: hypoxia/reoxygenation.

Haloperidol (Hal) (Figure 1A), a classical anti-psychotic butyrophenone drug, has been shown to possess vasodilatory and cardioprotective effects. Hal at a clinical dose relived symptoms of unstable angina pectoris and ameliorated ischemic changes seen on electrocardiography in patients [11], but large-sample studies are hampered by its extrapyramidal adverse reaction. Therefore, we used the piperidine group of Hal to modify and screen 1 compound, *N*-n-butyl haloperidol iodide (F_2_) (Figure 1B), which was found to maintain the relaxation of the coronary artery but have no extrapyramidal side reactions [12]. The compound has been patented. Our previous studies have shown that F_2_ has protective effects on *in vivo* myocardial I/R injury and *in vitro* H/R injury models [13, 14]. Its cardioprotective mechanism might be associated with the inhibition of calcium overload by blocking ventricular myocyte calcium channels and suppressing parameter. Recently, we found that F_2_ could ameliorate H/R-induced apoptosis [15]. In this study, we used a well-established H/R injury model that causes cardiomyocyte death in the H9c2 culture line, and tested the hypothesis that the protective effects of F_2_ are associated with inhibiting autophagy to reduce cardiomyocyte apoptosis.

## RESULTS

### F_2_ alleviates hypoxia/reoxygenation injury

We assessed cell viability in every group via MTT assay. F_2_ (10^−5^-10^−7^ mol/L) ameliorated cell viability in a concentration dependent manner (Figure 1C). Since lactate dehydrogenase (LDH) leakage is widely used as a marker of cellular damage, cardiomyocyte cells injury was assessed by determining LDH activity in culture medium at the end of reoxygenation. LDH leakage increased in the H/R group compared with the control group, but was significantly decreased by F_2_ treatment (Figure 1D). These findings indicated that F_2_ could promote cell survival and reduce cell damage in H9c2 cells subjected to H/R.

### F_2_-mediated protection involves inhibition of autophagy in cardiomyocytes following H/R

**Figure 2:**
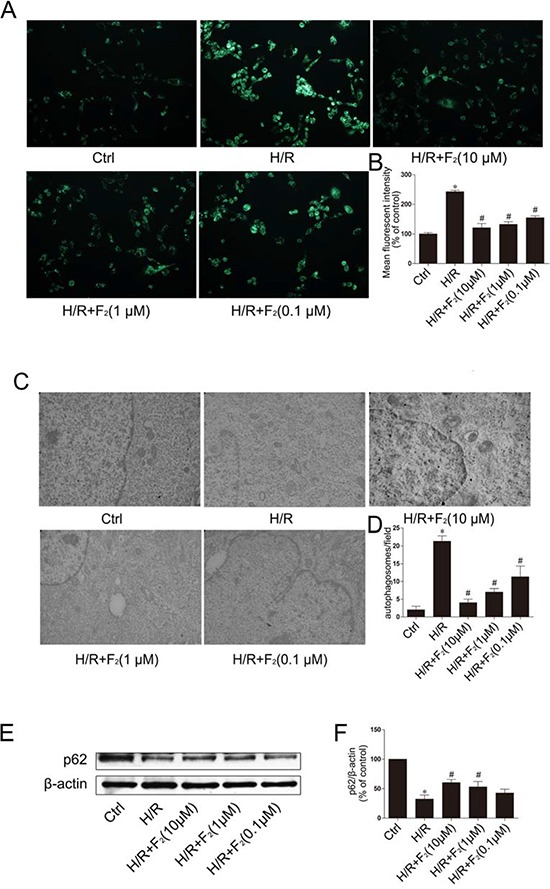
Effect of F_2_ on H/R-induced autophagy in H9c2 cells **A.** Autophagic vacuoles were stained with MDC. **B.** Quantification of mean fluorescent intensity in panel A. **C.** Ultrastructure features were examined by transmission electron microscopy (TEM), detected with magnification of × 25, 000. **D.** Quantification of the number of autophagosomes in panel C. **E.** Protein expression of p62. **F.** Quantification of panel E with densitometry. β-actin was used as a loading control. The data shown are represented as the means ± SD confirmed in three separate experiments. *p < 0.05 vs. control, #*p* < 0.05 vs. H/R. Ctrl: control; H/R: hypoxia/reoxygenation.

Activation of autophagy occurs in cardiomyocytes following H/R. To identify the role of F_2_ in regulating H/R-mediated autophagy in cardiomyocytes, we examined whether F_2_ could inhibit autophagy in cardiomyocytes, following H/R, by MDC staining and transmission electron microscopy (TEM). The autofluorescent substance MDC has been shown to be a specific marker for autophagic vacuoles (AVs). When cells are viewed with a fluorescence microscope, AVs stained by MDC appear as distinct dot-like structures distributed within the cytoplasm or localized to the perinuclear regions. In the H/R group, an increase in MDC-labeled vesicles was observed, as indicated by punctuate MDC fluorescence (Figure 2A and 2B), suggesting an induction of AV formation after H/R. In the F_2_-treated groups, the number of MDC-labeled vesicles declined in a dose-dependent manner. Autophagy was further confirmed by TEM. H9c2 cells after H/R showed typical autophagic vacuoles, including accumulation of numerous autophagic vesicles with a distinct double membrane, compared with no or few autophagic vacuoles in control cells. As above, F_2_ treatment reduced autophagic vacuoles in a dose-dependent manner (Figure 2C and 2D).

SQSTM1 (p62) is associated with mature autophagic vesicles and is degraded within autophagosomes. Western blot analysis revealed that p62 protein levels were reduced after H/R, and F_2_ treatment inhibited the reduction of p62 protein in a dose-dependent manner (Figure 2E and 2F).

### F_2_ inhibits the expression of autophagy markers in H9c2 cells subjected to H/R

**Figure 3:**
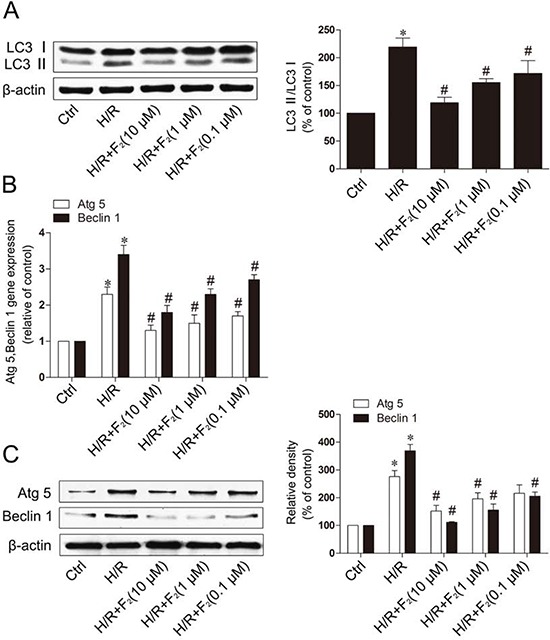
F_2_ inhibits the degree of autophagy in H9c2 cells subjected to H/R **A.** Western blot was performed with H9c2 cells for the expression of autophagic marker proteins LC3. **B.** The expression level of Atg5, Beclin 1 mRNA was analyzed with qRT-PCR. **C.** Western blot was analyzed the expression of Atg5, Beclin 1. β-actin was used as the loading control. Values are means ± SD. *p < 0.05 vs. control, #*p* < 0.05 vs. H/R. Ctrl: control; H/R: hypoxia/reoxygenation.

Microtubule-associated protein light chain 3 (LC3) is a specific marker for autophagy initiation. LC3-II is an accepted marker for autophagosome formation, although higher autophagosome accumulation may result from either increased autophagosome formation (autophagy initiation) or interrupted autophagosome degradation (autophagosome clearance). Western blot analysis revealed that LC3-II was up-regulated in H9c2 cells exposed to H/R (Figure 3A). And F_2_ could inhibit the expression of LC3-II in a dose-dependent manner. To further investigate the effect of F_2_ on autophagy, we used qRT-PCR and western blot to determine the expression levels of the autophagy-related genes, Atg5 and Beclin-1. Expression of Atg5 or Beclin-1 mRNA and protein were increased in H9c2 cells subjected to H/R, and F_2_ reduced the expression of Atg5 and Beclin-1 in a dose-dependent manner (Figure 3B and 3C). The above data clearly indicate that F_2_ could inhibit autophagy induced by H/R in H9c2 cells.

### F_2_ inhibits autophagy induced by H/R through the *MIF /mTOR* pathway

**Figure 4:**
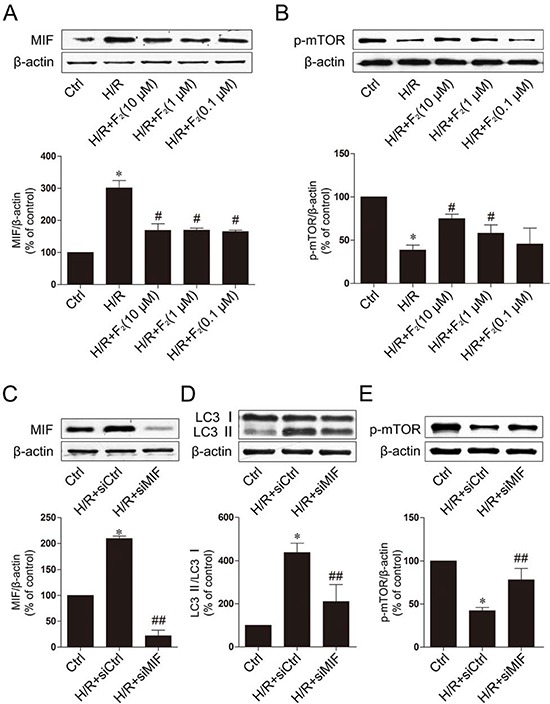
F_2_ inhibits autophagy through the *MIF-mTOR* pathway in H9c2 cells subjected to H/R **A.** Effect of F_2_ on the MIF expression in H9c2 cells subjected to H/R. **B.** Effect of F_2_ on the p-mTOR expression in H9c2 cells subjected to H/R. **C.** The knockdown efficiency was analyzed by western blot. **D.** Effect of knockdown MIF on the LC3 expression in H9c2 cells subjected to H/R. **E.** Effect of knockdown MIF on the p-mTOR expression in H9c2 cells subjected to H/R. The data shown are represented as the means ± SD confirmed in three separate experiments. *p < 0.05 vs. control, #*p* < 0.05 vs. H/R, ##*p* < 0.05 vs. H/R+siCtrl. Ctrl: control; H/R: hypoxia/reoxygenation.

We next investigated the molecular mechanism underlying of F_2_ inhibit H/R-induced autophagy. Recent evidence suggests an effect of MIF/mTOR signaling in the regulation of autophagy in murine cardiomyocytes. However, whether this signaling pathway is mediated by F_2_ in cardiomyocyte H/R has not been assessed. We first investigated whether F_2_ could regulate the MIF/mTOR signaling pathway in our H/R model. Western blot analysis demonstrated that the expression of MIF markedly increased and the phosphorylation of mTOR significantly decreased in cardiomyocytes following H/R injury (Figure 4A and 4B), demonstrating that F_2_ could significantly inhibit H/R-induced MIF. Reduced phosphorylation levels of mTOR were also observed to occur in a dose-dependent manner after F_2_ treatment compared to the H/R group.

To further investigate the function of MIF, we then knocked down MIF by using MIF-specific siRNA, the knockdown efficiency was shown in Figure 4C. Our results show that MIF knockdown significantly reduced autophagy after cardiomyocytes (Figure 4D). In addition, knockdown of MIF significantly increased the phosphorylation of mTOR after H/R (Figure 4E). Taken together, these findings indicate that F_2_ treatment-dependent recovery of cardiomyocytes from H/R may occur in part through the *MIF/mTOR* pathway to inhibit autophagy.

### H/R induced apoptosis is related to autophagy

**Figure 5:**
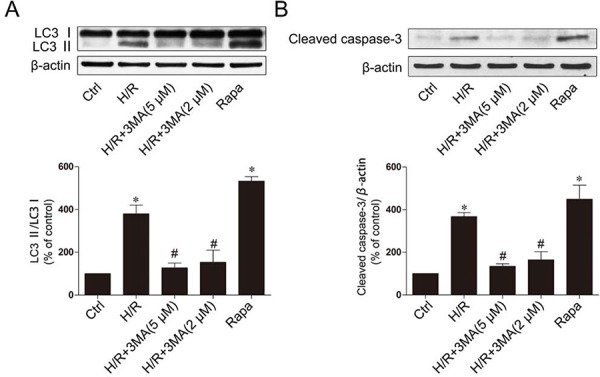
Inhibition of autophagy decrease apoptotic cell death **A.** The expression of LC3 with 3MA or Rapa. **B.** The expression of cleaved caspase-3 with 3MA or Rapa. H9c2 cells were treated with 100 ng/ml rapamycin. The data shown are represented as the means ± SD confirmed in three separate experiments. *p < 0.05 vs. control, #*p* < 0.05 vs. H/R. Ctrl: control; H/R: hypoxia/reoxygenation; Rapa, rapamycin.

To further confirm our hypothesis that inhibition of autophagy is a mechanism for the protective effect of F_2_ in H/R recovery, we used a classical autophagy inhibitor: 3-methyladenine (3-MA), a phosphoinositide 3-kinase inhibitor that exerts its autophagy-inhibiting effect before the formation of autophagosome, and a classical autophagy sensitizer: rapamycin, which is generally used to induce autophagy. The expression of LC3-II and cleaved caspase-3 were decreased by pretreatment with 3-MA in the H/R group, compared to the untreated H/R group (Figure 5A and 5B). Pretreatment with rapamycin significantly increased LC3- II and cleaved caspase-3 expression, indicating that rapamycin could induce apoptosis.

### F_2_ decreases H/R induced apoptosis in H9c2 cells

**Figure 6:**
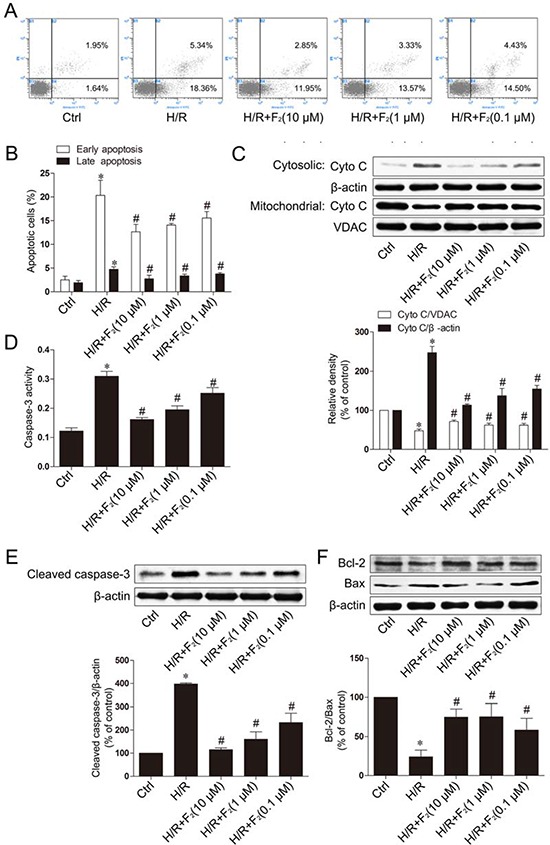
F_2_ protects cardiomyocytes against H/R-induced apoptosis **A.** Flow cytometry analysis of the annexin V-FITC/PI staining. **B.** Quantification histograms indicated the percentages of early (annexin V-FITC positive and PI negative) and late (annexin V-FITC positive and PI positive) apoptotic cells. **C.** Western blot analysis of cytochrome c in cytosolic and membrane fractions of H9c2 cells treated with F_2_ and exposed to hypoxia-reoxygenation. VDAC is a mitochondrial marker. **D.** Caspase-3 activity in lysates of H9c2 cells exposed to H/R with and without F_2_ treatment. **E.** Cleaved caspase-3 protein assayed by western blot at different treatment concentrations. **F.** Effects of F_2_ on apoptosis-related proteins assayed by western blot at different treatment concentrations. β-actin was probed as the loading control. Values are means ± SD. *p < 0.05 vs. control, #*p* < 0.05 vs. H/R. Ctrl: control; H/R: hypoxia/reoxygenation.

To determine the effect of F_2_ on apoptosis induced by H/R in H9c2 cells, we first examined early apoptosis by flow cytometry following annexin V-FITC and propidium iodide (PI) dual labeling. F_2_ (10^−5^-10^−7^ mol/L) ameliorated apoptosis induced by H/R in a concentration dependent manner. These results strongly demonstrate that F_2_ attenuates H/R-induced apoptosis in H9c2 cells (Figure 6A and 6B).

To further investigate the protection against apoptosis in H9c2 cells, we examined the mitochondrial cell death pathway and measured the release of cytochrome c from the inner mitochondrial space by western blot. F_2_ could inhibit H/R-induced cytochrome c release into the cytosolic fraction in a concentration dependent manner (Figure 6C). The release of cytochrome c from the mitochondria is a well-known mechanism to trigger downstream activation of caspase-9, which in turn cleaves caspase-3, resulting in cell death. An analysis of caspase-3 activity in the lysates of H9c2 cells revealed that F_2_ treatment reduced H/R-induced caspase-3 activity in a dose-dependent manner following H/R, compared with H9c2 cells not treated with F_2_ (Figure 6D). To further illustrate F_2_ could ameliorate apoptosis in H9c2 cells subjected to H/R, we performed western blot analysis for the apoptotic marker cleaved caspase-3 (Figure 6E). The results show that the cleaved forms of caspase-3 bands were dramatically induced when subjected to H/R, and F_2_ could inhibit cleaved caspase-3 expression. To further confirm the protective effects of F_2_ against H/R-induced apoptosis, the expression levels of apoptosis-related proteins such as Bcl-2 and Bax were examined using western blot analysis. The Bcl-2/Bax ratio decreased in the H9c2 cells exposed to H/R and this decrease was greatly attenuated by F_2_ treatment (Figure 6F).

## DISCUSSION

Many patients suffer from ischemic heart disease secondary to acute myocardial infarction. These are among the most prevalent health problems worldwide and are a major cause of morbidity and mortality. After ischemia, a long period of secondary myocardial injury occurs following myocardial reperfusion, including oxidative stress, inflammation, necrosis and apoptosis. Loss of myocardial cells is the main factor that interferes with recovery from the secondary damage. A series of previous studies have shown that F_2_ has protective effects on myocardial I/R injury *in vivo* and in *in vitro* H/R injury models [13–18]. However, as a calcium antagonists, the protective effect of F_2_ on the autophagy-induced cell death observed in cardiomyocyte H/R is not yet fully understood. In our previous study, we showed that F_2_-mediated protection is associated with antagonizing intracellular calcium overload through L-type calcium channels and suppressing damage. In the present study, we use a cardiomyocyte H/R model to demonstrate that F_2_ exerts a protective effect against H/R-induced injury, and that the molecular mechanism involves F_2_-mediated inhibition of autophagy. This data not only identify a novel mechanism of F_2_, but also provides impetus for the research and development of novel calcium antagonists for therapeutic use in the treatment of cardiac disease. The next significant work should focus on exploring the relevance and mechanism of calcium channels and autophagy.

Macrophage migration inhibitory factor (MIF), a pleiotropic cytokine, facilitates inflammation and modulates metabolism. MIF is believed to control the inflammatory “set point” during responses to a variety of inflammatory stimuli. MIF regulates inflammatory cell activation and release of other pro-inflammatory cytokines [19] and its pathological role has been reported in various inflammatory disorders, such as rheumatoid arthritis, atherosclerosis and cardiovascular diseases [20, 21]. MIF promotes prolonged I/R injury by enhancing the inflammatory response [22]. However, some evidence has suggested cardioprotective roles of MIF under various pathological conditions, including type 1 diabetes [23] and ischemia-reperfusion [24–26]. Several scenarios have been depicted for MIF-induced biological responses, including activation of the AMPK and JNK signaling cascades. More interestingly, a recent study revealed that MIF could inhibit autophagy [27–29]. In our study, knockdown MIF partially inhibited autophagy in cardiomyocyte H/R (Figure 4D). The mammalian target of rapamycin (mTOR) is a serine/threonine protein kinase that regulates cell growth, cell proliferation, cell motility, cell survival, protein synthesis, and transcription. mTOR belongs to the phosphatidylinositol 3-kinase-related kinase protein family. As a central protein of the PI3K family, mTOR is an upstream signal and plays an important role in the regulation of autophagy. Moreover, downstream signaling of MIF/mTOR plays an important role in programmed cell death, and phosphorylated mTOR provides cardioprotection by reducing autophagy and enhancing recovery in myocardial I/R [30]. As a macrolide antibiotic, rapamycin is often used to induce autophagy via mTOR inhibition [31]. The mTOR-signaling pathway is considered a master regulator of multiple interrelated functions and mechanisms relevant to cell growth, proliferation and death. Cardiac mTOR overexpression is sufficient to provide substantial cardioprotection against I/R injury and suppress the inflammatory response [32]. Furthermore, mTOR may directly modulate separate mechanisms controlling myocardial cell death, such as mechanisms involving the pro-apoptotic molecules BAD and Bcl-2 [33]. This dual role of mTOR may be based on the level of autophagy activation and/or various activated phases or situations. The mTOR pathway may have opposing effects on myocardial cell death mechanisms, which depend on the situation and stress level. These factors must be considered when designing potential therapies. In this study, we observed that autophagy is excessively activated during the early stages of H/R, and F_2_ treatment reduces the expression of MIF (Figure 4A) and further enhances mTOR signaling (Figure 4B), resulting in autophagy inhibition. Taken together, these findings suggest that the role of F_2_ in cardiomyocyte death and recovery from H/R involves inhibition of autophagy via the MIF/mTOR signaling pathway (Supplementary Figure 1).

Autophagy is an important intracellular bulk degradation process, whereby cytosolic proteins and organelles are sequestered by double membrane vesicles termed autophagosomes and delivered to lysosomes for degradation [34]. Autophagy occurs at basal levels, thereby participating in homeostatic functions in cells, but can be further induced by stress, such as nutrient depletion and oxidative stress during I/R. Autophagy has been implicated in myocardial I/R, although the exact functional role of autophagy in cell survival and death pathways associated with heart damage remains unclear. Several lines of evidence suggest that autophagy may promote cell survival by purging the cell of damaged proteins to generate the intracellular building blocks required to maintain vital functions during nutrient-limiting condition [5, 35]. Autophagy may also promote cell death through excessive self-digestion and degradation of essential cellular constituents or it may interact with the apoptotic cascade in the heart [36], indicating a double-edged sword for the biological function of autophagy in myocardial I/R injury. Several lines of evidence suggest that autophagy may promote cell apoptosis, and inhibition of the autophagy could decrease apoptosis [4, 8]. The present study showed increased the expression of cleaved caspase-3 protein (Figure 5B), and up-regulation of LC3- II (Figure 3A) and Beclin-1 levels (Figure 3B and 3C) in the cardiomyocytes H/R model, which suggest that H/R could induce autophagy and apoptosis. After F_2_ treatment, the induction of the LC3-II protein decreased significantly after cardiomyocyte H/R (Figure 3A). The effect of F_2_ was consistent with that of the classical autophagy inhibitor, 3-MA. Conversely, rapamycin-induced autophagy in cardiomyocytes could induce apoptosis (Figure 5A and 5B), which confirms that autophagy-induced apoptosis is a primary event in the secondary damage of in myocardial I/R. To the best of our knowledge, this is the first study demonstrating that F_2_ protects cardiomyocytes from H/R injury via the inhibition of excessive autophagy.

In conclusion, F_2_ significantly reduces the extent of damage following H/R injury, and improves the survival of cardiomyocytes. This protective role of F_2_ is related to inhibition of excessive autophagy. Furthermore, activation of the downstream signaling pathways MIF/mTOR is essential for the effect of F_2_ in myocardial cell death. Our study demonstrates that therapeutic strategies using F_2_ may be suitable for recovery from myocardial I/R injury.

## MATERIAL AND METHODS

### Materials and antibodies

*N*-n-butyl haloperidol iodide (F_2_) was synthesized in our laboratory. Caspase-3 assay kits, HEPES, 3-(4,5-dimethylthiazol-2yl)-2,5-diphenyltetrazo-lium bromide (MTT) and dimethyl sulfoxide (DMSO) were purchased from Sigma (St. Louis, MO, USA). F_2_ was dissolved in DMSO. The final DMSO concentration in solution was ≤ 0.1%, and the concentration had no effect on viability of normal H9c2 cells or H/R-induced H9c2 cells. Antibodies against cleaved caspase-3, LC3, SQSTM1 (P62), cytochrome C and Bax were purchased from Cell Signaling Technology (Danvers, MA, USA). Antibodies against MIF were purchased from Abcam (Cambridge, UK). Antibodies against phospho-mTOR, Beclin-1, Bcl-2, and Atg5 were purchased from Santa Cruz Biotechnology (Santa Cruz, CA, USA). Antibodies against β-actin, anti-rabbit secondary antibody, and anti-mouse secondary antibody were purchased from Wuhan Boster Biotechnology Limited Company (Wuhan, China). Dulbecco's modified Eagle's medium (DMEM) and fetal bovine serum (FBS) were purchased from Gibco Laboratories (Carlsbad, CA, USA). The annexin V-FITC Apoptosis Detection kit was purchased from Dojindo Molecular technologies, Inc. (Shanghai, China). Lipofectamine™ 2000 were obtained from Invitrogen Biotechnology (Camarillo, USA). SiRNAs were obtained from Shanghai Genepharma Co., Ltd. (Shanghai, China). All other chemicals and regents were purchased from local agencies.

### H9c2 cell culture

H9c2 cardiomyocytes derived from rat myocardium were obtained from the American Type Culture Collection (ATCC, Rockville, MD). H9c2 cells were cultured in DMEM supplemented with 10% FBS, 100 μ/mL penicillin, and 100 μ/mL streptomycin and maintained in a humidified atmosphere of 5% CO_2_ at 37°C. The medium was changed every 2 days. All assays contained appropriate controls, were performed in triplicate, and repeated on three separately initiated cultures.

### Hypoxia/Reoxygenation protocol

Hypoxia was induced by replacing the initial culture medium with buffer pH 6.2: 137 mM NaCl, 12 mM KCl, 0.49 mM MgCl_2_ · 6H_2_O, 0.9 mM CaCl_2_, 4 Mm HEPES, and 20 mM Na lactate [37]. H9c2 cardiomyocytes were incubated in an air-tight chamber gassed with pure N_2_ at 37°C for 1 h. Following exposure to hypoxia, the buffer was then replaced with fresh oxygenated culture medium and H9c2 cells were reoxygenated for 3 h in a humidified tissue culture incubator with 5% CO_2_-95% air.

### Assessment of H9c2 cell viability by MTT assay

The viability of H9c2 cardiomyocytes was measured using an MTT Cell Proliferation Assay Kit according to the manufacturer's instructions [38]. After H/R treatment, MTT was added to incubate for 4 h at 37°C and solubilized with 150 μL DMSO. The absorbance was measured by a microplate reader at 490 nm. Cell viability was measured as a percent calculated as follows: Viability (%) = (OD of Assay − OD of Blank) / (OD of control − OD of blank) × 100%.

### Assay of LDH activity

To determine the amount of cell injury induced by H/R protocol, LDH activities in the culture media were measured using a Lactate Dehydrogenase Activity Assay kit (Jiancheng Bioengineering Institure, Nanjing, China) according to the manufacturer's directions.

### Monodansylcadaverine (MDC) staining

MDC is a specific maker for autophagic vacuoles. H9c2 cells were inoculated in 24-well plates with sterile cover slips. Control H9c2 cells and those subjected to H/R in the presence or absence of F_2_ were incubated with 0.05 mM MDC (Sigma, USA) in fresh medium for 30 min at 37°C in the dark. After incubation, cells were washed three times with PBS and immediately analyzed with a fluorescence microscope.

### Transmission electron microscope (TEM)

Transmission electron microscopy was used to detect cellular ultrastructure. H9c2 cells were collected by centrifugation and washed three times with PBS, then fixed in glutaraldehyde (2.5% w/v) in 0.1 M cacodylate buffer overnight. After rinsing with buffer, the cells were then fixed in Karnofsky's fixative, post-fixed in 1% osmium tetroxide for 15 min, rinsed in water, and stained in aqueous uranyl acetate (3% w/v) for 30 min. Cell monolayers were then scraped, pelleted, and examined with a transmission electron microscope.

### Mitochondrial and cytosolic fractionation

To determine the release of pro-apoptotic factors from mitochondria to cytosol by western blot, we isolated mitochondria using a Mitochondria Isolation Kit (Beyotime Institute of Biotechnology, China). Briefly, cells were incubated in 100 μL ice-cold mitochondrial lysis buffer on ice for 10 min. Cell suspension was then taken into a glass homogenizer and homogenized for 30 strokes using a tight pestle on ice. The homogenate was subjected to centrifugation at 600 g for 10 min at 4°C to remove nuclei and unbroken cells. Then the supernatant was collected and centrifuged again at 12000 g for 30 min at 4°C to obtain the cytosolic (supernatant) and mitochondrial (deposition) fraction. Samples of cytosol and mitochondria were dissolved in lysis buffer and proteins were subjected to western blot for the analysis of cytochrome c (cyto C).

### RNA extraction and quantitative real-time polymerase chain reaction (qRT-PCR)

The expression level of Atg5 and Beclin 1 genes were determined by qRT-PCR. Briefly, total RNA was extracted from H9c2 cells with RNAiso Plus according to the protocol of the manufacturer (Takara, Dalian, China). 500 ng of total RNA was used for generating cDNA by using a Primescript RT reagent kit from Takara. Resulting cDNAs were quantified by real-time PCR on an ABI 7500 Real-Time PCR System (Applied Biosystems) using the following primers: Atg5: 5′-AGTGGAGGCAACAGAACC-3′ (sense) and 5′-GACACGAACTGGCACATT-3′ (antisense); Beclin 1: 5′-GGCAGTGGCTCCTATT-3′ (sense) and 5′-GGCGTGCTGTGCTCTGAAAA-3′. Data was normalized to β-actin transcript levels using 5′-CTGTATGCCTCTGGTCGTAC-3′ (sense) and 5′-TGATGTCACGCACGATTTCC-3′ (antisense) primers. Expression was calculated using ΔΔC_t_ method.

### Transfection of MIF small interfering RNA (siRNA)

The cells were inoculated in 6-well plates and transfected with siRNA, using Opti-MEM media (Gibco by life Technologies). Briefly, 5 μL of MIF siRNA (20 μM) was mixed with Opti-MEM media. Separately, Lipofectamine™ 2000 (Invitrogen) was mixed with Opti-MEM and then the mixtures were combined for 20 min at room temperature. The mixture was then added to each well containing cells and medium and incubated for 6 h. Medium was then changed into antibiotic-free DMEM supplemented with 10% FBS for 48 h. MIF siRNA duplexes (sense, GUCGGGAGAAAUAAAUGGUdTdT; antisense, ACCAUUUAUUUCUCCCGACdTdT) or control nonspecific siRNA duplexes (sense, CGUUUGUUCGCUUCCUGAGTT; antisense, CUCAGGAAGCGAACAAACGTG).

### Flow cytometry

For assessment of apoptosis, an annexin V-FITC/PI staining kit (Dojindo Molecular technologies) was used according to the manufacturer's protocol. Briefly, H9c2 cells were washed with phosphate-buffered saline (PBS), then resuspended in 100 μL of binding buffer at 1 × 10^6^ cells/mL. Aliquots of 10 μL annexin V-FITC stock solution and 5 μL propidium iodide (PI) were added to the cells, and cells were incubated in the dark for 15 min room temperature. Immediately after mixing with 400 μL of binding buffer, the samples were analyzed by flow cytometry with use of a FACSort Flow Cytometer (Becton-Dickinson, Franklin Lake, NJ, USA). Approximately 10,000 cells were counted in each of the samples, and data were analyzed by use of WinMDI software (v2.9, bio-soft Net).

### Caspase-3 activity

Caspase-3 activity was assessed by a commercially available caspase-3 assay kit from Sigma, according to the manufacturer's protocol. Briefly, after lysis, the Bradford method was used to determine protein concentration. Supernatants were incubated with 2 mM caspase-3 substrate (Ac-DEVD-pNA) at 37°C for 1 h, and then the absorbance at 405 nm was measured with a spectrophotometer. Caspase-3 enzymatic activity in cell lysates was directly proportional to the color reaction.

### Western blot analysis

H9c2 cells were treated with the conditions as indicated, cells were harvested, washed three times with ice-cold PBS and lysed in ice-cold RIPA lysis buffer for 30 min. For western blot analysis after protein quantification with a BCA assay kit (PIERCE, Rockford, USA). Equal amounts of denatured protein samples were subjected to SDS-PAGE and probed with the corresponding antibodies as indicated at 4°C overnight, followed by incubation with a 1:5,000 dilution of HRP-conjugated secondary antibody for 1 h at room temperature. The transferred proteins were visualized with a SurperSignal West Dura detection kit (Pierce Biotech, Rockford, USA) and exposed to Medical Blue X-ray film. Results were analyzed with Gel-pro software by densitometric analysis (Media Cybernetics, USA).

### Statistical analysis

All assays were repeated at least three times independently. All data were expressed as the mean± standard deviation (S.D) and analyzed with SPSS 13.0 statistical software (SPSS Inc.). Differences between groups were determined with a one-way analysis of variance (ANOVA) followed by a Student-Newman-Keuls test. *P* < 0.05 was considered statistically significant.

## SUPPLEMENTARY FIGURE



## Abbreviations

3-MA: 3-methyladenine
ATG: autophagy-related gene
Cyto C: Cytochrome C
DMSO: dimethyl sulfoxide
F_2_: *N*-n-butyl haloperidol iodide
H/R: hypoxia/reoxygenation
I/R: ischemia/reperfusion
LC3: microtubule associated protein light chain 3
MDC: monodansylcadaverine
MIF: Macrophage migration inhibitory factor
mTOR: mammalian target of rapamycin
MTT: 3-(4,5-dimethylthiazol-2-yl)-2,5-diphenyltetrazolium bromide
Rapa: rapamycin
siRNA: Small interfering RNA
SQSTM1: sequestosome 1
