# Supplementary material for: N-n-butyl haloperidol iodide protects cardiomyocytes against hypoxia/reoxygenation injury by inhibiting autophagy
Source: Oncotarget. 2015 Sep 2;6(28):24709–21. doi: 10.18632/oncotarget.5077 (PMC4694790; doi:10.18632/oncotarget.5077)
Supplement: Supplementary file 1 [file oncotarget-06-24709-s001.pdf]

SUPPLEMENTARY FIGURE

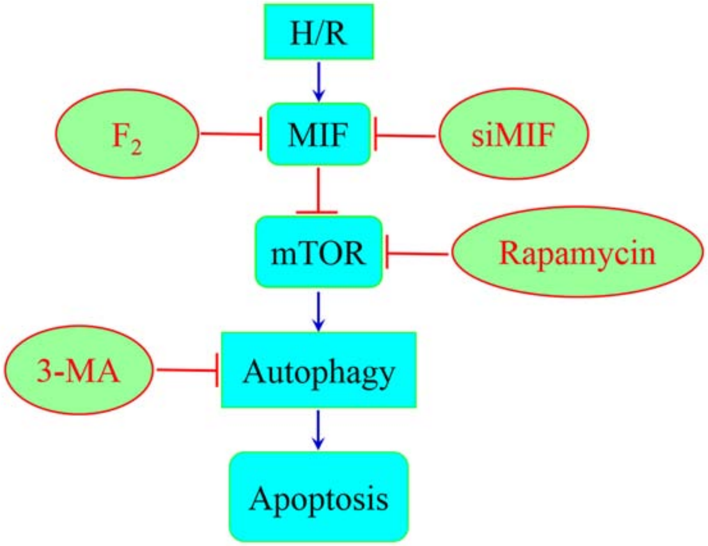

Supplementary Figure S1: Schematic representation of the proposed model for F_2 inhibited H/R-induced autophagy in H9c2 cells.
